# Effectiveness and safety of meropenem–vaborbactam versus ceftazidime–avibactam in multidrug-resistant Gram-negative infections: a systematic review and meta-analysis with trial sequential analysis

**DOI:** 10.1128/aac.01546-25

**Published:** 2026-01-06

**Authors:** Shahd Mohammad, Yamama Al Namer, Wafaa Rahimeh, Mosab Albalas, Thamer A. Almangour

**Affiliations:** 1 Clinical Pharmacy Department, Sheikh Khalifa Medical City 37532, Abu Dhabi, United Arab Emirates; 2 Pharmacy Department, Sheikh Tahnoon Bin Mohammad Medical City, Abu Dhabi, United Arab Emirates; 3 Clinical Pharmacy Department, College of Pharmacy, Al Ain University, Abu Dhabi, United Arab Emirates; 4 Clinical Pharmacy Department, Sheikh Tahnoon Bin Mohammad Medical City, Abu Dhabi, United Arab Emirates; 5 Clinical Pharmacy Department, College of Pharmacy, King Saud University 37850, Riyadh, Saudi Arabia; University of Pennsylvania Perelman School of Medicine, Philadelphia, Pennsylvania, USA

**Keywords:** meta-analysis, β-lactam/β-lactamase inhibitors, carbapenem-resistant enterobacterales, ceftazidime–avibactam, meropenem–vaborbactam

## Abstract

Antimicrobial resistance driven by multidrug-resistant (MDR) Gram-negative pathogens poses a major global threat, contributing to substantial morbidity and mortality. Novel β-lactam/β-lactamase inhibitor combinations, particularly meropenem–vaborbactam (M/V) and ceftazidime–avibactam (C/A), have expanded therapeutic options; however, their comparative efficacy and safety remain uncertain. This meta-analysis compared M/V and C/A in adult patients with MDR Gram-negative infections. MEDLINE, Embase, and Cochrane Central were searched for studies evaluating M/V versus C/A in hospitalized adults. Outcomes included all-cause mortality, clinical cure, and microbiological recurrence; safety was assessed qualitatively. Data were synthesized using Review Manager, with trial sequential analysis (TSA) applied to minimize random error. Five retrospective cohort studies (three full articles and two conference abstracts) comprising 3,280 patients were included, of whom 577 received M/V and 2,703 received C/A. Populations predominantly consisted of older adults aged 57–70 years, with respiratory tract infections being most common. Pooled analyses demonstrated no statistically significant differences between M/V compared to C/A in all-cause mortality (Odds ratio [OR] 0.87; 95% CI 0.69–1.11; *P* = 0.26; I² = 16%), clinical cure (OR 1.41; 95% CI 0.66–3.03; *P* = 0.37; I² = 55%), and microbiological recurrence (OR 0.67; 95% CI 0.32–1.40; *P* = 0.29; I² = 0%). Qualitative synthesis indicated comparable tolerability. TSA for mortality demonstrated insufficient evidence for definitive conclusions. M/V showed no statistically significant difference over C/A; therefore, selection should be guided judiciously based on clinical context. Further studies are needed to define the optimal role of each agent within antimicrobial stewardship frameworks.

## INTRODUCTION

Antimicrobial resistance represents one of the most pressing challenges in modern medicine, driving high mortality, prolonged hospitalization, and escalating healthcare costs worldwide (1–3). This crisis is particularly pronounced among multidrug-resistant (MDR) Gram-negative pathogens, for which therapeutic options are severely restricted, and clinical outcomes remain poor. Among these, carbapenem-resistant *Enterobacterales* (CRE) have emerged as pathogens of critical concern. Resistance in CRE arises from a constellation of mechanisms, including porin loss, efflux pump upregulation, and production of carbapenem-hydrolyzing β-lactamases (4, 5). These synergistic mechanisms compromise the efficacy of last-line carbapenems and are associated with unfavorable outcomes (6, 7).

In response to this therapeutic challenge, novel β-lactam/β-lactamase inhibitor (BL/BLI) combinations have been developed, substantially broadening the antimicrobial armamentarium against MDR Gram-negative organisms. Ceftazidime–avibactam (C/A) received U.S. Food and Drug Administration approval in 2015, followed by meropenem–vaborbactam (M/V) in 2017 (8, 9). Both agents exhibit robust *in vitro* activity against CRE and are endorsed by the Infectious Diseases Society of America (IDSA) as preferred treatment options for infections caused by CRE (10, 11). Important distinctions exist between the two agents. First, avibactam and vaborbactam inhibit different carbapenemase classes. Second, in M/V, the β-lactam to β-lactamase inhibitor ratio is 1:1, whereas it is 4:1 in C/A, reflecting differences in pharmacokinetic/pharmacodynamic target attainment. Third, the meropenem backbone in M/V provides broader penicillin-binding protein (PBP)-binding affinity and anaerobic activity compared with ceftazidime (12, 13). These pharmacologic and mechanistic differences raise the possibility of divergent clinical outcomes.

In light of these considerations, the comparative efficacy and safety of M/V versus C/A have attracted increasing attention. However, the available evidence remains heterogeneous and at times conflicting, leaving uncertainty as to whether M/V offers superior outcomes relative to C/A. To address this critical gap, we conducted a systematic review and meta-analysis evaluating the efficacy and safety of M/V compared with C/A in adult patients with MDR Gram-negative infections.

## MATERIALS AND METHODS

This systematic review and meta-analysis were performed and reported in accordance with the Cochrane Collaboration Handbook for Systematic Review of Interventions (14) and the Preferred Reporting Items for Systematic Reviews and Meta-Analysis (PRISMA) Statement guidelines (15). As such, the protocol was prospectively registered with the International Prospective Register of Systematic Reviews (PROSPERO) database under registration number CRD420251137797. There was no requirement for informed consent or Institutional Review Board approval for this meta-analysis, given that the data are publicly available and we did not have access to individual patient data.

### Eligibility criteria

Inclusion in this systematic review was restricted to studies that met all of the following criteria: (i) randomized controlled trials or non-randomized studies, whether prospective or retrospective; (ii) directly compared M/V with C/A; (iii) enrolled adult patients hospitalized with MDR Gram-negative infections, including CRE; and (iv) reported at least one relevant clinical outcome of interest. Studies were excluded if they evaluated either agent independently without direct comparison, failed to report clinical outcomes, or involved overlapping populations. To minimize publication bias, conference abstracts were considered eligible if the above criteria were satisfied. In such cases, corresponding authors were contacted for supplementary data, and abstracts were included only when adequate information for analysis was available.

### Search strategy and data extraction

We systematically searched MEDLINE through PubMed, Embase, and Cochrane Central from inception to September 2025. No filters or language restrictions were applied. The detailed search strategies for each database are provided in Table S1. Additionally, we searched https://clinicaltrials.gov and performed a backward snowballing search of the reference lists of included studies and relevant reviews to identify additional eligible records. Two reviewers (Y.A. and W.R.) collaboratively developed and independently conducted the database search and study selection. Any discrepancies were resolved through discussion, and when consensus could not be reached, a third reviewer (S.M.) adjudicated. Data extraction was subsequently performed by (S.M.) using a piloted, standardized data collection form, structured in alignment with the review objectives and clinical focus.

### Endpoints and subgroup analyses

The outcomes assessed were (i) all-cause mortality, defined as death at any time point reported in the included studies; (ii) clinical cure, defined as resolution of infection-related signs and symptoms as reported by study investigators or inferred from the absence of clinical failure; (iii) microbiological recurrence or relapse, defined as re-isolation of the causative pathogen following documented eradication; and (iv) adverse events, as specified by study-defined criteria. Subgroup analyses were conducted to evaluate mortality stratified by confirmed CRE infections and by *Klebsiella pneumoniae* carbapenemase (KPC)-producing CRE isolates.

### Quality assessment

We evaluated the risk of bias using the Newcastle–Ottawa scale. The assessment was independently conducted by two authors (Y.A. and W.R.). Disagreements were resolved through discussion until a consensus was reached. Publication bias was investigated by funnel plot analysis of point estimates in relation to study weights.

### Statistical analysis

Odds ratios (ORs) with 95% confidence intervals (CIs) were used to compare treatment effects for categorical outcomes. Heterogeneity was evaluated using the I² statistic and Cochran’s Q test; *P* <0.10 or I² >40% was considered indicative of significant heterogeneity. A random-effects model with the restricted maximum likelihood method was applied. Leave-one-out sensitivity analyses were conducted to assess the robustness of pooled estimates. Review Manager 5.4 (The Cochrane Collaboration, Copenhagen, Denmark) was used for statistical analysis.

### Trial sequential analysis

Trial sequential analysis (TSA) was performed using TSA software version 0.9.5.10 (Copenhagen Trial Unit, Denmark) to reduce the risk of random errors and to calculate the required information size. The dichotomous outcome assessed was all-cause mortality. Event rates were extracted as proportions when available; outcomes not reported in this format were excluded. Analyses were conducted with a two-sided α of 0.05 and 80% power. The required information size was estimated based on observed event rates and variance within the M/V and C/A groups. Monitoring boundaries were applied to interpret results: crossing superiority or non-inferiority boundaries confirmed an effect, crossing futility boundaries indicated no difference, and failure to reach the required information size without boundary crossing indicated that the evidence remains inconclusive.

## RESULTS

### Study selection and baseline characteristics

**Fig 1:**
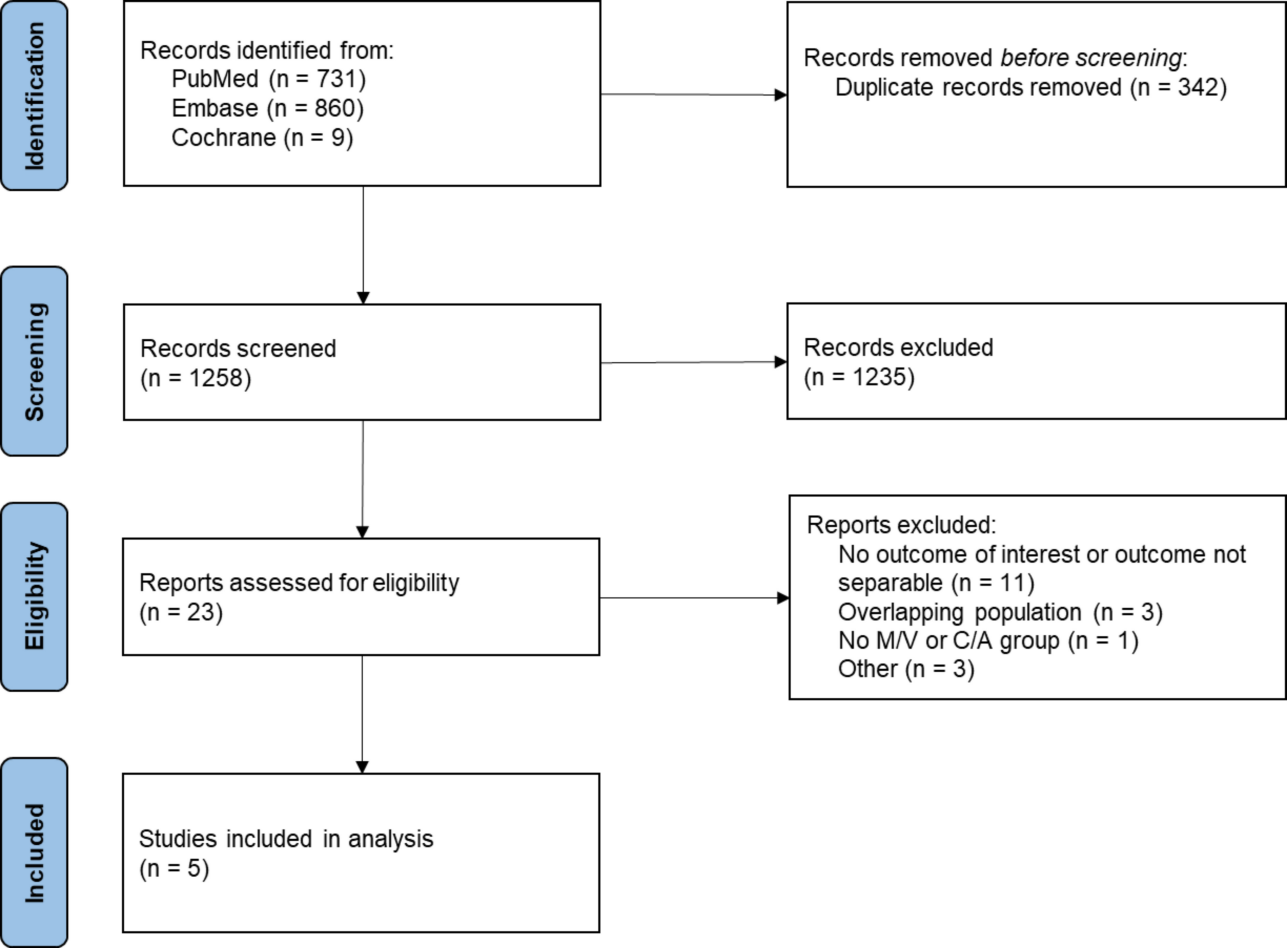
PRISMA flow diagram. M/V, meropenem/vaborbactam; C/A, ceftazidime/avibactam.

The initial search yielded 1,600 records. After removal of duplicates and screening of titles and abstracts, 1,235 records were excluded, primarily because they comprised microbiological surveillance or epidemiological studies, pharmacokinetic/pharmacodynamic investigations, *in vitro* susceptibility studies, or literature reviews that did not meet the inclusion criteria. Three articles and two conference abstracts were ultimately included, comprising a total of 3,280 patients, all derived from retrospective cohort studies. No randomized controlled trials meeting the inclusion criteria were identified (Fig. 1). Reasons for exclusion of assessed records are detailed in Table S2.

Of the 3,280 patients included in the meta-analysis, 577 (17.6%) received M/V and 2,703 (82.4%) received C/A. Across studies, patients were predominantly older adults, with ages ranging from 57 to 70 years. Most cohorts were male, with a weighted mean male proportion of 58.8%. Respiratory tract infections were the most common site, accounting for 52.2% (1,625/3,112) of cases, followed by urinary tract infections at 15.6% (474/3,024), based on cohorts where the site of infection was reported. *Klebsiella pneumoniae* was the predominant pathogen, identified in 75.4% (381/505) of reported cases, while the KPC resistance gene was detected in 78.4% (396/505) of isolates among cohorts with available molecular data (Table 1).

**TABLE 1:** Study characteristics^*a*^

	Marino, 2025 (16)		Zilberberg, 2025 (17)		Ackley, 2020 (18)		Karaba, 2024 (19)		Mezzadri, 2024 (20)	
	M/V (*n* = 36)	C/A (*n* = 52)	M/V (*n* = 455)	C/A (*n* = 2,320)	M/V (*n* = 26)	C/A (*n* = 105)	M/V (*n* = 23)	C/A (*n* = 95)	M/V (*n* = 37)	C/A (*n* = 131)
Study design	Retrospective cohort—PSM		Retrospective cohort		Retrospective cohort		Retrospective cohort—IPW		Retrospective cohort	
Country	Italy		US		US		US		Italy	
Publication type	Original article		Original article		Original article		Conference abstract		Conference abstract	
Age, years^*b*^	61^b^	62^b^	62.2^a^	60.8^a^	57.5^b^	62^b^	58^b^	63^b^	70^b^	68^b^
Male, %	52.8	55.8	56	60.6	46.2	55.2	52.2	63.2	62.2	62.6
Charlson comorbidity index^*b*^	NR	NR	3.6^a^	3.5^a^	5^b^	5^b^	NR	NR	5^b^	5^b^
APACHE II score^*b*^	17^b^	18^b^	NR	NR	27^b^	26^b^	NR	NR	NR	NR
ICU admission, %	100	100	47.7	50.9	65.4	55.2	67	61.3	NR	NR
Mechanical ventilation, %	83.3	73.1	35.0	41.4	NR	NR	56.5	42.1	NR	NR
Vasopressors, %	NR	NR	34.1	35.7	NR	NR	52.2	51.6	NR	NR
Sepsis, %	86.1	90.4	27.7	25.2	NR	NR	NR	NR	NR	NR
Septic shock, %	63.9	59.6	45.1	43.5	NR	NR	NR	NR	NR	NR
Site of infection										
BSI, %	61.1	50	NR	NR	34.6	41.9	34	34.9	NR	NR
UTI, %	NI	NI	19.6	14.1	3.8	12.3	12.2	15.4	NR	NR
RTI, %	19.4	11.5	48.1	56.8	46.2	35.2	17.4	23.2	NR	NR
IAI, %	NI	NI	4.6	4	30.8	17.1	4.4	8.4	NR	NR
SSTI, %	NI	NI	NR	NR	23.1	19.0	26.1	16.8	NR	NR
Causative pathogen										
*Escherichia coli*, %	NI	NI	NR	NR	11.5	8.6	7.8	12.8	0	NR
*Klebsiella pneumoniae,* %	100	100	NR	NR	57.7	72.4	31	30.9	100	98.5
*Enterobacter cloacae*, %	NI	NI	NR	NR	30.8	19.1	9.9	15.3	0	NR
Polymicrobial infection, %	19.4	21.2	NR	NR	57.7	59.1	NR	NR	NR	NR
KPC resistance gene, %	100	100	NR	NR	76.9	71.9	73.3	64	73	82.2
Hospital acquired, %	91.7	90.4	NR	NR	50	61.9	NR	NR	NR	NR
Combination, %	16.7	55.8	NR	NR	15.4	61	NR	NR	NR	NR
Duration of treatment, days	10^b^	10.5^b^	NR	NR	12.3^b^	10.8	14^b^	14^b^	NR	NR

^*a*^ M/V, meropenem/vaborbactam; C/A, ceftazidime/avibactam; PSM, propensity score matched; IPW, inverse probability weighted; BSI, bloodstream infection; UTI, urinary tract infection; RTI, respiratory tract infection; IAI, intra-abdominal infection; SSTI, skin and soft tissue infection; ICU, intensive care unit; NR, not reported; NI, not included; KPC*, Klebsiella pneumoniae* carbapenemase.

^*b*^ Data presented as mean or median as available. a: mean; b: median.

### Pooled analyses of all included studies

**Fig 2:**
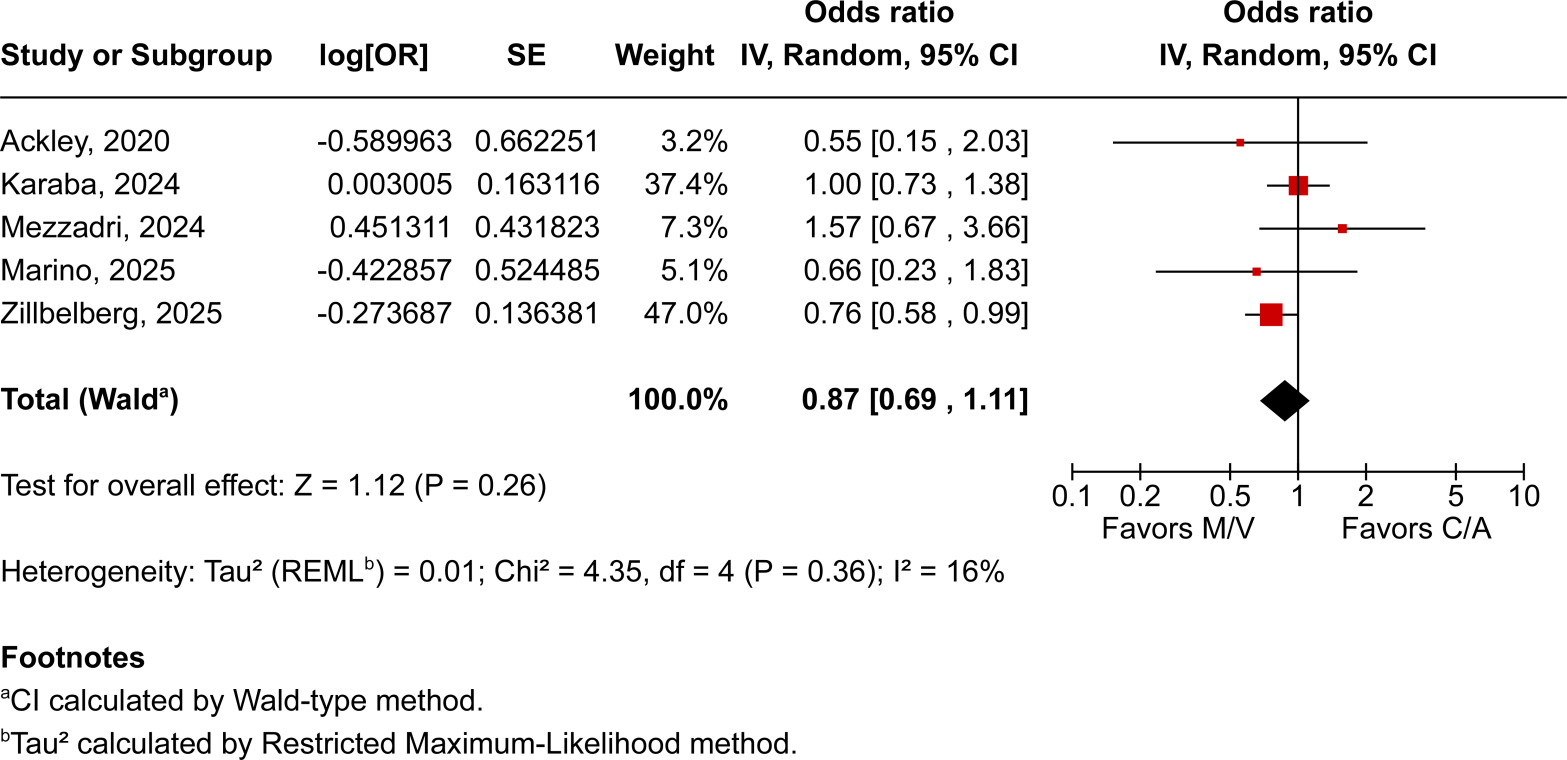
All-cause mortality between patients receiving M/V compared to those receiving C/A.

**Fig 3:**
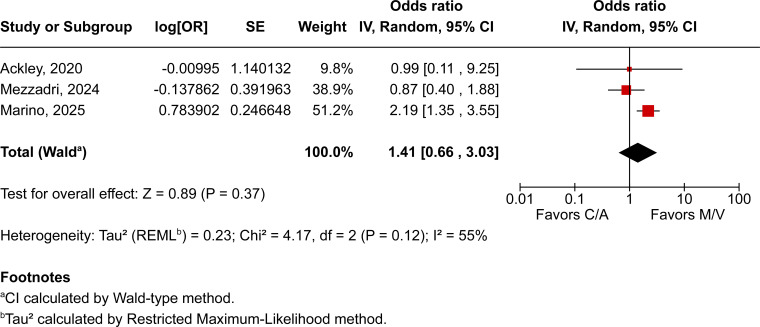
Clinical cure rates among patients treated with M/V compared with C/A; moderate heterogeneity was observed (I² = 55%).

**Fig 4:**
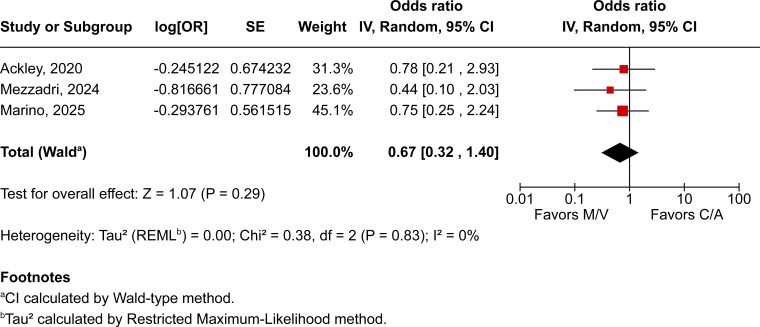
Microbiological recurrence among patients treated with M/V compared with C/A.

Across all included patients, pooled analyses demonstrated no statistically significant differences between those treated with M/V over those receiving C/A across all evaluated outcomes. All-cause mortality was comparable between groups (OR 0.87; 95% CI 0.69–1.11; *P* = 0.26; I² = 16%; Fig. 2), as were clinical cure (OR 1.41; 95% CI 0.66–3.03; *P* = 0.37; I² = 55%; Fig. 3) and microbiological recurrence (OR 0.67; 95% CI 0.32–1.40; *P* = 0.29; I² = 0%; Fig. 4).

### Subgroup analysis

When restricted to the four studies that exclusively enrolled patients with confirmed CRE infections, pooled results remained consistent, showing no statistically significant difference in mortality between M/V over C/A (OR 0.99; 95% CI 0.75–1.31; *P* = 0.96; I² = 0%; Fig. S1). Additionally, an exploratory subgroup analysis limited to KPC-producing CRE isolates, derived from the two studies reporting genotype-stratified data, likewise demonstrated no significant difference in mortality between M/V over C/A (OR 0.97; 95% CI 0.81–1.15; *P* = 0.72; I² = 0%; Fig. S2).

### Sensitivity analysis

Leave-one-out sensitivity analyses demonstrated robust results, with pooled ORs for all-cause mortality ranging from 0.79 to 0.99 and corresponding I² values between 0% and 25%. Notably, exclusion of the two conference abstracts resulted in a statistically significant pooled effect favoring M/V (OR 0.74; 95% CI 0.58–0.96; *P* = 0.02; I² = 0%; Fig. S3). For clinical cure, ORs ranged from 0.88 to 2.11 with heterogeneity values between 0% and 75%. For microbiological recurrence or relapse, ORs ranged from 0.61 to 0.76, with I² consistently at 0%.

### Safety outcome

Safety outcomes were inconsistently reported across the five included studies, precluding quantitative pooling; therefore, findings are summarized qualitatively. Nephrotoxicity was evaluated in two studies. Ackley et al. (18), applying AKIN criteria, reported nephrotoxicity in 14.3% of patients treated with M/V compared with 29.2% of those receiving C/A, with no statistically significant difference between groups (*P* = 0.16). Similarly, Zilberberg et al. (17) observed acute kidney injury (AKI) in 53.9% of patients treated with M/V versus 56.6% of those receiving C/A; however, the definition of AKI was not specified, and the difference was not significant (*P* = 0.28). When limited to AKI not present on admission, the rates were 17.6% for M/V and 19.4% for C/A, again without statistical significance (*P* = 0.38). *Clostridioides difficile* infection was also assessed in two studies. Marino et al. (16) reported no cases among patients treated with M/V compared with 1.9% in the C/A group, while Zilberberg et al. (17) documented infection in 12.1% of M/V recipients versus 14.3% of those treated with C/A, with no significant difference (*P* = 0.29). Adjusted analyses yielded consistent findings, showing no statistically significant difference between groups (*P* = 0.12).

Other adverse events were reported only by Ackley et al. (18), who found similar rates of leukopenia, rash, and neurotoxicity between M/V- and C/A-treated patients, with no statistically significant differences (*P* = 1.00). Among the two conference abstracts, Mezzadri et al. (20) stated that adverse events were comparable between groups without providing numerical data, whereas Karaba et al. (19) did not report any safety outcomes.

### Trial sequential analysis

TSA was conducted for the outcome of all-cause mortality using predefined monitoring boundaries (α = 0.05, power = 80%). Of the five studies included in the meta-analysis, four were eligible for TSA, as Karaba et al. (19) reported outcomes exclusively as adjusted odds ratios without raw event data, precluding inclusion. As illustrated in Fig. S4, the analysis demonstrated that the cumulative Z-curve did not cross either the conventional significance threshold or the TSA-adjusted monitoring boundaries for benefit, harm, or futility. The accrued information size (*n* = 3,162) did not reach the required information size of 3,647 patients (~87%), indicating that the available evidence remains inconclusive and that additional studies are necessary to achieve sufficient statistical power for definitive conclusions. TSA for other outcomes, including clinical cure and microbiological recurrence, was not feasible due to the limited number of eligible studies.

### Quality assessment

The Newcastle–Ottawa scale was used to assess the quality of all included studies. The three original articles (16–18) were assessed as having a low risk of bias. For the two conference abstracts (19, 20), several domains could not be adequately evaluated due to insufficient methodological information and the inability to obtain further details from the authors; therefore, these were judged to have a high risk of bias, as summarized in Table S3. Funnel plot inspection revealed a symmetrical distribution of studies around the pooled effect estimate, with no evident asymmetry (Fig. S5).

## DISCUSSION

In this systematic review and meta-analysis of five studies involving patients with MDR Gram-negative infections, we assessed the comparative efficacy and safety of M/V versus C/A. The pooled analyses revealed no statistically significant differences between treatment arms with respect to all-cause mortality, clinical cure, or microbiological recurrence. Safety outcomes were synthesized qualitatively due to heterogeneous reporting; nevertheless, the incidence of AKI and *Clostridioides difficile* infection did not differ significantly between M/V and C/A treated patients across reporting studies. TSA indicated that additional studies are required to achieve sufficient statistical power for definitive conclusions.

MDR Gram-negative infections impose a substantial clinical and economic burden, with CRE representing the most critical threat due to their association with high mortality and limited therapeutic options (1–3). Carbapenem resistance arises through multiple mechanisms, most notably the production of carbapenemases, including class A such as KPC, class B such as New Delhi metallo-β-lactamase (NDM), and class D such as OXA-48-like enzymes, or through non-carbapenemase pathways, including overexpression of extended-spectrum β-lactamases (ESBLs) or class C AmpC β-lactamases in combination with porin loss or efflux pump upregulation (21, 22). Carbapenemase production is the most clinically significant mechanism, conferring broad resistance and often coexisting with other determinants. Historically, treatment relied on polymyxin-based regimens; yet outcomes have been poor, with lower clinical cure, higher mortality, and markedly increased nephrotoxicity compared with newer agents (23). These limitations have prompted a paradigm shift toward the use of novel BL/BLI combinations, such as C/A, M/V, and imipenem–relebactam (I/R), which pair potent β-lactams with structurally advanced inhibitors capable of restoring β-lactam activity through inhibition of key carbapenemases, offering superior stability and potency compared with earlier inhibitors (24).

Among the BL/BLI combinations, C/A and M/V represent the most extensively utilized agents for the management of CRE infections, whereas I/R remains less extensively evaluated. C/A, approved in 2015, couples the established antipseudomonal cephalosporin ceftazidime with avibactam, a diazabicyclooctane β-lactamase inhibitor that confers potent inactivation of class A, class C, and select class D carbapenemases, thereby restoring activity against KPC- and OXA-48-producing *Enterobacterales* (25, 26). Conversely, M/V, approved in 2017, combines meropenem, an intrinsically broad-spectrum carbapenem with stability against ESBL and AmpC β-lactamases, with vaborbactam, a cyclic boronic acid inhibitor that provides potent and sustained inhibition of KPC, as well as AmpC enzymes (9). Despite these mechanistic overlaps, important pharmacological distinctions arise from the intrinsic properties and BL:BLI dosing ratios of each combination. M/V employs a 1:1 ratio, reflecting the closely aligned pharmacokinetics of meropenem and vaborbactam and the need to meet two separate PK/PD targets—%fT > MIC for meropenem and fAUC/MIC for vaborbactam. C/A, by contrast, uses a 4:1 ratio, designed simply to keep avibactam above its minimal protective concentration while ceftazidime drives bacterial killing, resulting in an overall PK/PD profile that largely mirrors ceftazidime once avibactam exposure is adequate (12). Beyond ratio effects, both inhibitors lack activity against NDM; however, vaborbactam additionally does not inhibit OXA-48 but offers greater stability against KPC hydrolysis than avibactam (24, 27). M/V also demonstrates higher epithelial lining fluid and peritoneal penetration, and unlike C/A, retains anaerobic activity through the meropenem backbone (28).

Current IDSA guidance reflects these pharmacological and *in vitro* distinctions but emphasizes the lack of definitive comparative clinical evidence between M/V and C/A. The IDSA guidance does not favor one agent over the other for CRE-associated complicated urinary tract infections, acknowledging insufficient high-quality data. For non-urinary tract CRE infections, recommendations are stratified by carbapenemase type: M/V is preferentially recommended for KPC-producing isolates, largely based on data from a single cohort study and *in vitro* studies, whereas C/A is favored for OXA-48-producing strains, with the guidance recommending against M/V in this setting. In difficult-to-treat *Pseudomonas aeruginosa*, C/A is likewise preferred, with recommendations against M/V (11).

In this context, our meta-analysis was framed broadly to assess M/V versus C/A across all MDR Gram-negative infections, and the pooled estimates did not demonstrate a statistically significant mortality benefit with M/V. Moreover, restricting the analysis to studies that enrolled patients with confirmed CRE, excluding Zilberberg et al. (17), which lacked pathogen-level data, yielded non-significant findings. This finding may be explained, at least in part, by clinical and pharmacological factors intrinsic to the included populations. The site of infection represents a major determinant of BL/BLI efficacy. In particular, respiratory tract infections have been repeatedly associated with decreased rate of C/A clinical success (29, 30), likely attributable to suboptimal epithelial lining fluid penetration, which may result in insufficient pharmacodynamic target attainment in the pulmonary compartment (31). In contrast, M/V has demonstrated favorable pharmacokinetic–pharmacodynamic properties in this setting. Data from the TANGO II trial (32), as well as subsequent cohort studies and *in vitro* models, suggest promising outcomes in respiratory tract infections, supported by substantial epithelial lining fluid penetration of both meropenem and vaborbactam (epithelial lining fluid/plasma concentration ratios of 65% and 79%, respectively) (33, 34). Within the studies included in our analysis, however, a relatively high proportion of patients treated with C/A had respiratory tract infections (56.8% in Zilberberg et al. [17], 35.2% in Ackley et al. [18], and 23.2% in Karaba et al. [19]), which may have influenced outcomes unfavorably for C/A in these cohorts and potentially obscured true differences between regimens. Indication-specific outcome analysis was limited by sparse reporting across studies. Only Marino et al. (16) provided site-stratified mortality estimates, with prematching hazard ratios of 0.82 (95% CI 0.18–3.70) for pneumonia and 0.88 (95% CI 0.30–2.53) for bloodstream infections when comparing M/V with C/A, indicating no statistically significant difference between the two regimens within either infection site. Post-matching interpretation was precluded by few events and small subgroup sizes. In addition to the infection site, baseline severity of illness likely influenced mortality outcomes. Predictors of poor prognosis with M/V, such as septic shock at infection onset and elevated Charlson comorbidity index (CCI ≥ 3) (35), were prevalent across the included studies. Reported mean CCI values exceeded 3 in all evaluable cohorts (5.0 in Ackley et al. [18], 5.0 in Mezzadri et al. [20], and 3.6 in Zilberberg et al. [17]), and septic shock was present in a substantial proportion of patients (63.9% in Marino et al. [16] and 45.1% in Zilberberg et al. [17]). These factors may have attenuated observable differences in mortality between the treatment groups, as both agents were frequently administered in populations with advanced illness and high baseline risk of death.

Notably, the sensitivity analysis excluding the two conference abstracts demonstrated a statistically significant mortality difference favoring M/V over C/A. This finding should still be interpreted with caution, given the limited evidence base of the remaining studies. Rather than altering clinical conclusions, this signal underscores the need for higher-quality comparative data to determine whether it reflects a true therapeutic advantage.

Another critical consideration involves infections caused by KPC-producing *Enterobacterales*, which represent the most prevalent carbapenemase mechanism worldwide and a major driver of therapeutic failure in CRE (36). Extensive *in vitro* evidence supports the enhanced activity of M/V against KPC producers, demonstrating consistently lower minimum inhibitory concentrations and higher susceptibility rates relative to alternative agents, including C/A (37, 38). Moreover, specific Ω-loop mutations within the bla_KPC_ gene (e.g., D179Y) have been shown to markedly reduce avibactam binding affinity, thereby conferring resistance to C/A, while exerting minimal impact on vaborbactam activity, further reinforcing the pharmacologic rationale for M/V preference in this setting (39). Nevertheless, our exploratory analysis of the two studies reporting mortality stratified by KPC status (Karaba et al. [19] and Marino et al. [16]) did not demonstrate a mortality benefit for M/V over C/A in infections caused by KPC-producing isolates. These findings should be interpreted cautiously, given the very limited genotype-specific data. Similarly, comparative assessment of resistance emergence between treatment arms was not feasible due to limited reporting across studies. Only Ackley et al. (18) provided relevant data, observing no emergence of resistance in the M/V group compared with 20% in the C/A group among patients with recurrent CRE infections, a difference that did not reach statistical significance (*P* = 1.00). Marino et al. (16), in contrast, excluded isolates resistant to either study drug at baseline, precluding evaluation of incident resistance.

Finally, infusion modality is an important determinant of BL/BLI pharmacokinetics and pharmacodynamics. Prolonged infusion improves PK/PD target attainment for time-dependent β-lactams, particularly in critically ill patients and deep-seated infections, and may reduce resistance emergence (40–42). However, evaluation of this factor in our meta-analysis was not feasible due to limited reporting. Only Marino et al. (16) provided infusion-stratified outcomes, and across all infusion approaches, M/V and C/A demonstrated no statistically significant differences in 30-day mortality: continuous infusion (HR 1.04, 95% CI 0.38–2.85), extended infusion (HR 0.70, 95% CI 0.24–2.04), and intermittent infusion (HR 0.81, 95% CI 0.09–1.31). Accordingly, no firm conclusions regarding the impact of infusion modality can be drawn.

Several prior meta-analyses have evaluated the role of M/V in CRE infections, though none have directly compared it with C/A. Bucataru et al. (43) synthesized four studies comparing M/V with best available therapy, which included polymyxins, aminoglycosides, tigecycline, carbapenems, C/A, or piperacillin–tazobactam. Their findings revealed no superiority of M/V for clinical cure at test-of-cure (83.1% vs 72.5%; RR 1.29; 95% CI 0.92–1.80; *P* = 0.14; I² = 68%) or at end-of-treatment (93% vs 89%; RR 1.11; 95% CI 0.92–1.33; *P* = 0.29; I² = 85%), results directionally concordant with our analysis, which demonstrated a non-significant trend favoring M/V in terms of clinical cure (*P* = 0.14). Notably, heterogeneity in clinical cure definitions likely contributed to variability across analyses: Marino et al. (16) defined cure by composite biochemical and clinical resolution (vasopressor discontinuation, reduction in procalcitonin or CRP, fever defervescence) assessed early in therapy, whereas Ackley et al. (18) defined cure at end of treatment based on leukocyte normalization, fever resolution, or physician clinical judgment. This subjectivity underscores the challenge of interpreting clinical cure across heterogeneous studies.

Interestingly, Bucataru et al. (43) did report a statistically significant reduction in 28-day all-cause mortality with M/V compared to best available therapy (3.1% vs 7.8%; RR 0.47; 95% CI 0.24–0.92; *P* = 0.03; I² = 0). This contrasts with our findings, in which no significant mortality difference was observed between M/V and C/A. Several factors may account for this discrepancy. First, the mortality benefit of novel BL/BLI agents over polymyxins and aminoglycosides is well established and underpins current guideline recommendations; thus, the inclusion of these older agents in best available therapy comparators likely amplified the relative advantage of M/V. Second, Bucataru et al. (43) included the Ackley et al. (18) cohort, where C/A served as the comparator into their analysis, with this single study contributing the greatest statistical weight (34.8%) to the mortality outcome. This may have driven the observed significance in the absence of sensitivity analyses to assess the robustness of the results. In contrast, the same study accounted for only 3.2% of the mortality analysis in our direct comparison of M/V versus C/A, limiting its impact on the pooled estimate. With respect to safety, Bucataru et al. (43) reported no significant difference in adverse events between M/V and best available therapy (35.2% vs 36.3%; RR 0.79; 95% CI 0.53–1.17; *P* = 0.23; I² = 53%). While our analysis could not quantitatively assess adverse events due to heterogeneous reporting, qualitative synthesis similarly suggested comparable safety profiles between M/V and C/A, consistent with real-world pharmacovigilance data demonstrating markedly lower nephrotoxicity compared with polymyxin-based regimens. Complementary evidence from Jin et al. (44), who conducted a single-arm meta-analysis of six studies, further supports the favorable efficacy and tolerability of M/V, reporting pooled clinical success rates of 75% (95% CI 66%–82%), 30-day survival of 75% (95% CI 71%–78%), and 90-day survival of 69% (95% CI 61%–76%), with serious adverse events infrequently encountered.

This meta-analysis constitutes the first comparative evaluation of M/V versus C/A in the treatment of MDR Gram-negative infections. In the absence of statistically significant differences across assessed clinical outcomes, therapeutic selection should be individualized, incorporating infection site, illness severity, patient-specific characteristics, and local resistance patterns rather than assuming inherent superiority of either regimen. From an antimicrobial stewardship standpoint, these findings underscore the importance of judiciously preserving novel BL/BLI agents for confirmed or strongly suspected CRE infections, reserving M/V for scenarios involving documented C/A resistance or intolerance to optimize therapeutic efficacy while minimizing selective resistance pressure. Integration of rapid genotypic diagnostic panel testing should be prioritized to facilitate timely and targeted therapy, particularly for accurate carbapenemase identification and in complex settings involving co-expression of resistance determinants (e.g., KPC with OXA-48), wherein C/A may offer broader coverage and represent the preferred option. Moreover, given that our TSA demonstrated that the cumulative sample size was underpowered to draw definitive conclusions for mortality outcomes, future research should focus on well-designed, adequately powered, genotype-stratified randomized controlled trials to validate these findings and further clarify the optimal clinical role of each agent.

This meta-analysis has several limitations. First, no randomized controlled trials were identified; all included studies were retrospective cohorts, which introduces potential selection bias despite adjustment through propensity score matching in some cohorts. Second, the absence of pathogen-level data in Zilberberg et al. (17) limited the ability to fully restrict analyses to confirmed CRE, although a subgroup analysis restricted to confirmed CRE yielded similar results. Third, genotype-specific outcome data were inconsistently reported. Although an exploratory analysis for KPC-producing isolates was conducted, interpretation should be approached with caution due to limited statistical power derived from only two contributing studies. Fourth, variability in clinical cure definitions and infection site distributions across cohorts may have contributed to interstudy heterogeneity. Fifth, inconsistent reporting of safety outcomes precluded quantitative synthesis. Sixth, data regarding antibiotic dosing strategies and minimum inhibitory concentrations were incomplete, precluding evaluation of their potential influence on clinical outcomes. Despite these limitations, we believe that our findings at least reflect the real-world setting.

### Conclusion

This meta-analysis found no statistically significant differences between M/V and C/A in mortality, clinical cure, or microbiological recurrence among patients with MDR Gram-negative infections. Both agents appear comparably effective and safe; therefore, therapeutic selection should be guided by illness severity, patient-specific characteristics, and local resistance patterns. Further well-designed, genotype-stratified randomized clinical trials are warranted to confirm these findings and define the optimal clinical role of each agent within antimicrobial stewardship frameworks.

## Data Availability

All data supporting the findings of this study are available within the manuscript. Additional details can be provided upon reasonable request.

## SUPPLEMENTAL MATERIAL

Supplemental material aac.01546-25-s0001.docx Tables S1 to S3; Fig. S1 to S5.

ASM does not own the copyrights to Supplemental Material that may be linked to, or accessed through, an article. The authors have granted ASM a non-exclusive, world-wide license to publish the Supplemental Material files. Please contact the corresponding author directly for reuse.
