# Supplementary material for: Effectiveness and safety of meropenem–vaborbactam versus ceftazidime–avibactam in multidrug-resistant Gram-negative infections: a systematic review and meta-analysis with trial sequential analysis
Source: Antimicrob Agents Chemother. 2026 Jan 6;70(2):e01546-25. doi: 10.1128/aac.01546-25 (PMC12888891; doi:10.1128/aac.01546-25)
Supplement: Supplemental material — Tables S1 to S3; Fig. S1 to S5. [file aac.01546-25-s0001.docx]

**Supplementary Material**

**Effectiveness and Safety of Meropenem–Vaborbactam vs Ceftazidime–Avibactam in Multidrug-Resistant Gram-Negative Infections: A Systematic Review and Meta-analysis with Trial Sequential Analysis**

Shahd Mohammad ^1*^, Yamama Al Namer ^2^, Wafaa Rahimeh ^3^, Mosab Albalas ^4^, Thamer A. Almangour ^5^

1. Clinical Pharmacy Department, Sheikh Khalifa Medical City, Abu Dhabi, United Arab Emirates
2. Pharmacy Department, Sheikh Tahnoon Bin Mohammad Medical City, Abu Dhabi, United Arab Emirates
3. Clinical Pharmacy Department, College of Pharmacy, Al Ain University, Abu Dhabi, United Arab Emirates
4. Clinical Pharmacy Department, Sheikh Tahnoon Bin Mohammad Medical City, Abu Dhabi, United Arab Emirates
5. Clinical Pharmacy Department, College of Pharmacy, King Saud University, Riyadh, Saudi Arabia

*Correspondence: [shamohammad@seha.ae](mailto:shamohammad@seha.ae)

**Supplementary Tables**

**Supplementary Table 1.** Detailed search strategy

| Database | Search strategy | Search Date | Coverage Dates | Filters applied | Language restriction |
| --- | --- | --- | --- | --- | --- |
| MEDLINE through PubMed | ("meropenem vaborbactam" OR Vabomere) AND ("avibactam ceftazidime" OR "ceftazidime avibactam" OR "Avycaz" OR "Zavicefta") | 01-September-2025 | From inception until September-2025 | None applied | None applied |
| Embase | ("meropenem vaborbactam" OR Vabomere) AND ("avibactam ceftazidime" OR "ceftazidime avibactam" OR "Avycaz" OR "Zavicefta") | 01-September-2025 | From inception until September-2025 | None applied | None applied |
| Cochrane | ("meropenem vaborbactam" OR Vabomere) AND ("avibactam ceftazidime" OR "ceftazidime avibactam" OR "Avycaz" OR "Zavicefta") | 01-September-2025 | From inception until September-2025 | None applied | None applied |

Note: No filters or language restrictions were applied. The search was conducted without limits on date, geography, or study design to ensure comprehensive retrieval.

**Supplementary Table 2.** Reason for exclusion after full text review

| **Study Title** | **Author, year** | **Study Design** | **Reason of Exclusion** |
| --- | --- | --- | --- |
| Clinical Outcomes of Patient Subgroups in the TANGOÂ II Study | Bhowmick, 2020 | RCT | Outcomes not separable for C/A |
| Clinical outcomes of serious infections due to carbapenem-resistant Enterobacteriaceae (CRE) in TANGO II, a phase 3, randomized, multi-national, open-label trial of meropenem-vaborbactam (M-V) vs. best available therapy (BAT) | Kaye, 2017 | RCT - Abstract | Outcomes not separable for C/A |
| COMPARATIVE EFFECTIVENESS OF MEROPENEM/VABORBACTAM VS CEFTAZIDIME/AVIBACTAM IN HOSPITAL PNEUMONIA | Zilberberg, 2024 | Retrospective Cohort - Conference Abstract | Overlapping population |
| Comparative Effectiveness of Meropenem/Vaborbactam vs. Ceftazidime/ Avibactam among Adults Hospitalized with an Infectious Syndrome in the US, 2019-2021 | Zilberberg, 2023 | Retrospective Cohort - Conference Abstract | Overlapping population |
| Comparison of ceftolozane/tazobactam, ceftazidime/avibactam, and meropenem/vaborbactam activity against P. Aeruginosa: A multicenter evaluation | Moise, 2020 | In-vitro susceptibility study | No Clinical Outcomes |
| Compassionate use of meropenem/vaborbactam for infections caused by KPC-producing Klebsiella pneumoniae: A multicentre study | Tumbarello, 2022 | Retrospective cohort | Non-comparator study |
| Cost-effectiveness analysis of vaborem for the treatment of carbapenem-resistant Enterobacteriaceae-Klebsiella pneumoniae carbapenemase (CRE-KPC) infections in the UK | Vlachaki, 2022 | Cost-effectiveness study | No Clinical Outcomes |
| Effect and Safety of Meropenem-Vaborbactam versus Best-Available Therapy in Patients with Carbapenem-Resistant Enterobacteriaceae Infections: the TANGO II Randomized Clinical Trial | Wunderink, 2018 | Trial Registration | Outcomes not separable for C/A |
| Effect and Safety of Meropenem–Vaborbactam versus Best-Available Therapy in Patients with Carbapenem-Resistant Enterobacteriaceae Infections: the TANGO II Randomized Clinical Trial | Wunderink, 2018 | RCT | Outcomes not separable for C/A |
| Extended-Infusion Î²-Lactam Therapy, Mortality, and Subsequent Antibiotic Resistance among Hospitalized Adults with Gram-Negative Bloodstream Infections | Karaba, 2024 | Retrospective cohort | Other: Irrelevant |
| Meropenem-vaborbactam vs standard of care for multidrug resistant carbapenem-resistant Enterobacteriaceae | Misikir, 2020 | Retrospective cohort | Outcomes not separable for C/A |
| Recurrence of infection and emergence of drug resistance after treatment with meropenem/vaborbactam compared with ceftazidime/avibactam in carbapenem-resistant Enterobacteriaceae infections | Ackley, 2019 | Retrospective cohort | Overlapping population |
| Temporal Trends in the Management and Mortality Associated With Klebsiella pneumoniae Carbapenemase-Producing Enterobacterales: A Cohort Study | Pinto, 2025 | Retrospective cohort | Outcomes not separable for M/V |
| The antibiotic de-escalation strategy in patients with multidrug-resistant bacterial colonization after allogeneic stem cell transplantation | Bono, 2025 | Retrospective cohort | Outcomes not separable |
| The prevalence of gram-negative bacteria with difficult-to-treat resistance and utilization of novel β-lactam antibiotics in the southeastern United States | Tsai, 2024 | Retrospective cohort | Other: Irrelevant |
| The use of new antibacterial drugs against infections caused by multidrug-resistant Gram-negative bacteria: an Italian real-world evidence study in a Lombardy hospital | Ferrara, 2024 | Retrospective cohort | Outcomes not separable for M/V |
| Utilization of Colistin Versus β-Lactam and β-Lactamase Inhibitor Agents in Relation to Acute Kidney Injury in Patients with Severe Gram-Negative Infections | Doremus, 2022 | Retrospective cohort | Outcomes not separable for M/V or C/A |
| Weighing the Odds: Novel β-Lactam/β-Lactamase Inhibitor Use in Hospital-Acquired and Ventilator-Associated Pseudomonas aeruginosa Pneumonia for Patients Who Are Morbidly Obese | Coyne, 2023 | Retrospective cohort | Other: Irrelevant |

**Supplementary Table 3.** Quality Assessment of Included Studies

| Author, Year | Selection | | | | Comparability | Outcome | | | Total Quality Score |
| --- | --- | --- | --- | --- | --- | --- | --- | --- | --- |
|  | Representativeness of the exposed cohort | Selection of the non-cohort | Ascertainment of exposure | The outcome of interest was not present at start of study | Comparability of cohorts | Assessment of outcome | Was follow-up long enough for outcomes to occur | Adequacy of follow up of cohorts |  |
| Marino, 2025 | 1 | 1 | 1 | 1 | 2 | 1 | 1 | 1 | 9 |
| Zillbelberg, 2025 | 1 | 1 | 1 | 1 | 2 | 1 | 1 | 1 | 9 |
| Ackley, 2020 | 1 | 1 | 1 | 1 | 2 | 1 | 1 | 1 | 9 |
| Karaba, 2024 | 1 | 1 | Insufficient data available to access this domain | 1 | 1 | 0 | Insufficient data available to access this domain | 0 | 4 |
| Mezzadri, 2024 | 1 | 1 | Insufficient data available to access this domain | 1 | Insufficient data available to access this domain | 0 | 0 | 0 | 3 |

**Supplementary Figure Legends**

**Supplementary Figure 1** All-cause mortality was not significantly different between M/V and C/A in patients with infections caused by Carbapenem-resistant Enterobacterales (CRE).

**Supplementary Figure 2** All-cause mortality was not significantly different between M/V and C/A in patients with infections caused by *Klebsiella pneumoniae* carbapenemase (KPC)–producing pathogens.

**Supplementary Figure 3** Sensitivity analysis for mortality excluding the two conference abstracts, demonstrating a statistically significant reduction in mortality among patients treated with M/V compared with C/A.

**Supplementary Figure 4** Trial sequential analysis (TSA) for all-cause mortality comparing M/V versus C/A using a two-sided α of 5% and 80% power. The cumulative Z-curve did not cross the conventional or TSA-adjusted monitoring boundaries, and the accrued information size (n = 3,162) did not reach the required information size (3,647), indicating insufficient evidence to confirm benefit, harm, or futility.

**Supplementary Figure 5** Funnel plot assessing publication bias for the outcome of mortality. The plot shows a symmetrical distribution of studies, suggesting low likelihood of publication bias.

**Supplementary Figures**

**
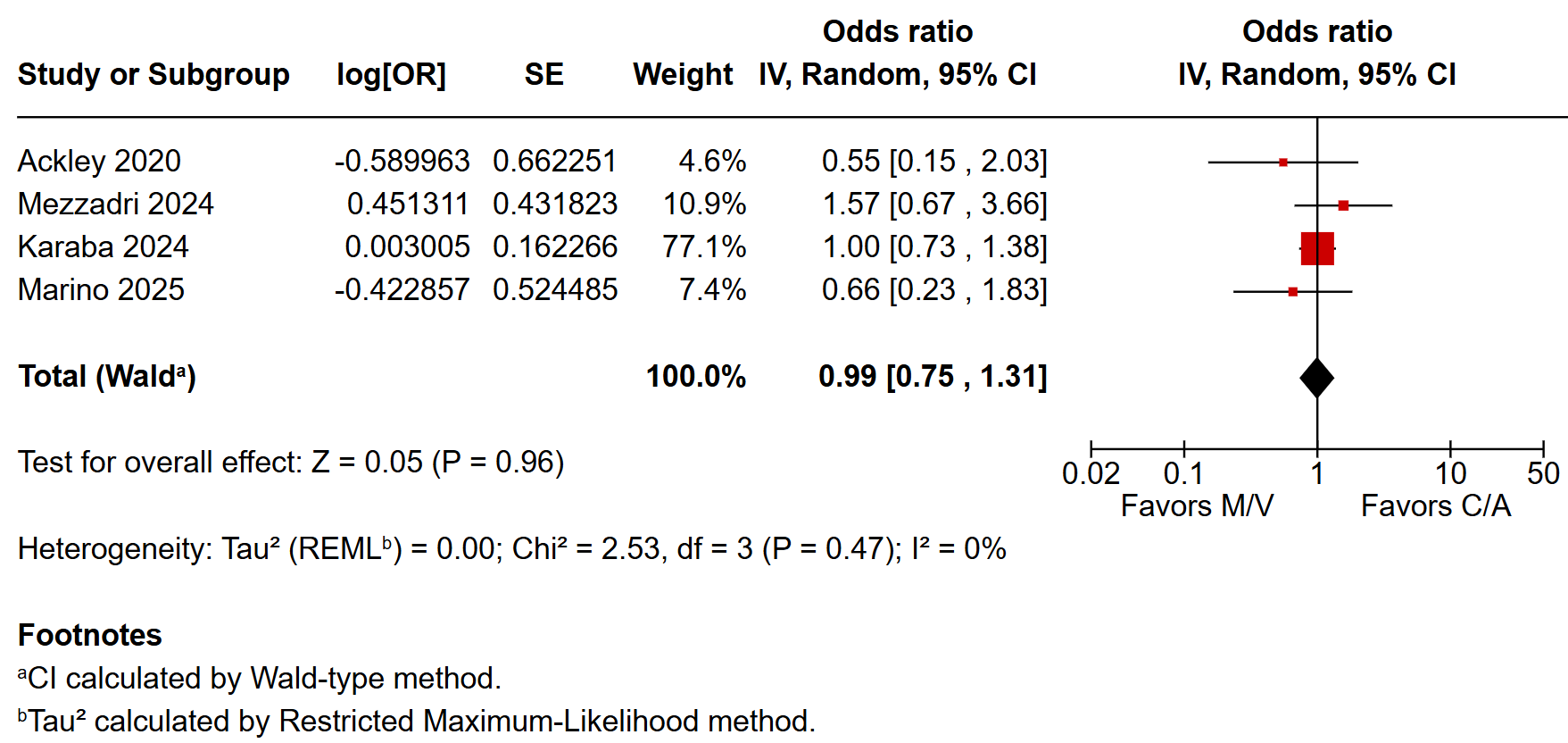
**

**Supplementary Figure 1**


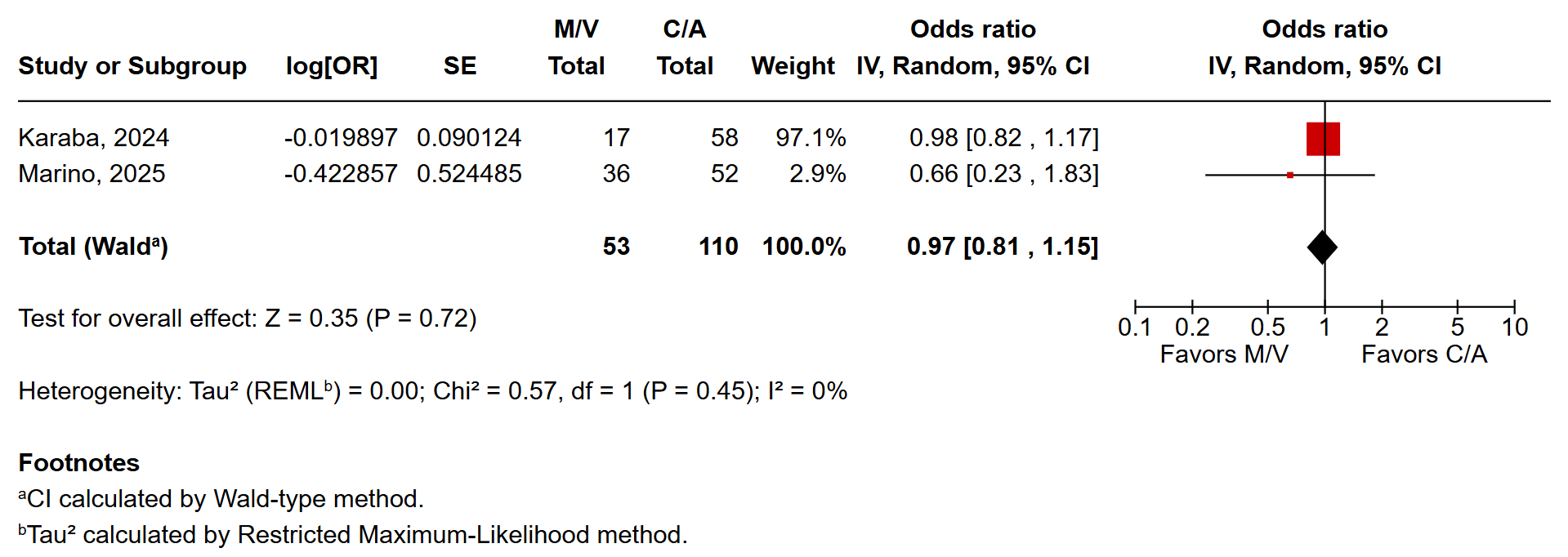


**Supplementary Figure 2**

**
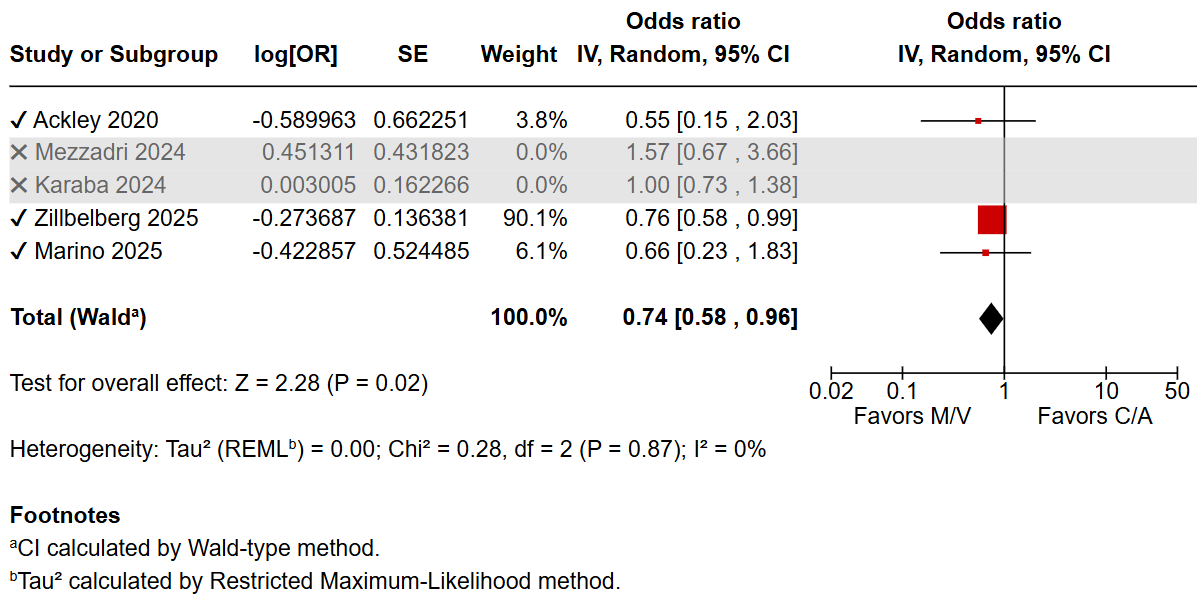
**

**Supplementary Figure 3**


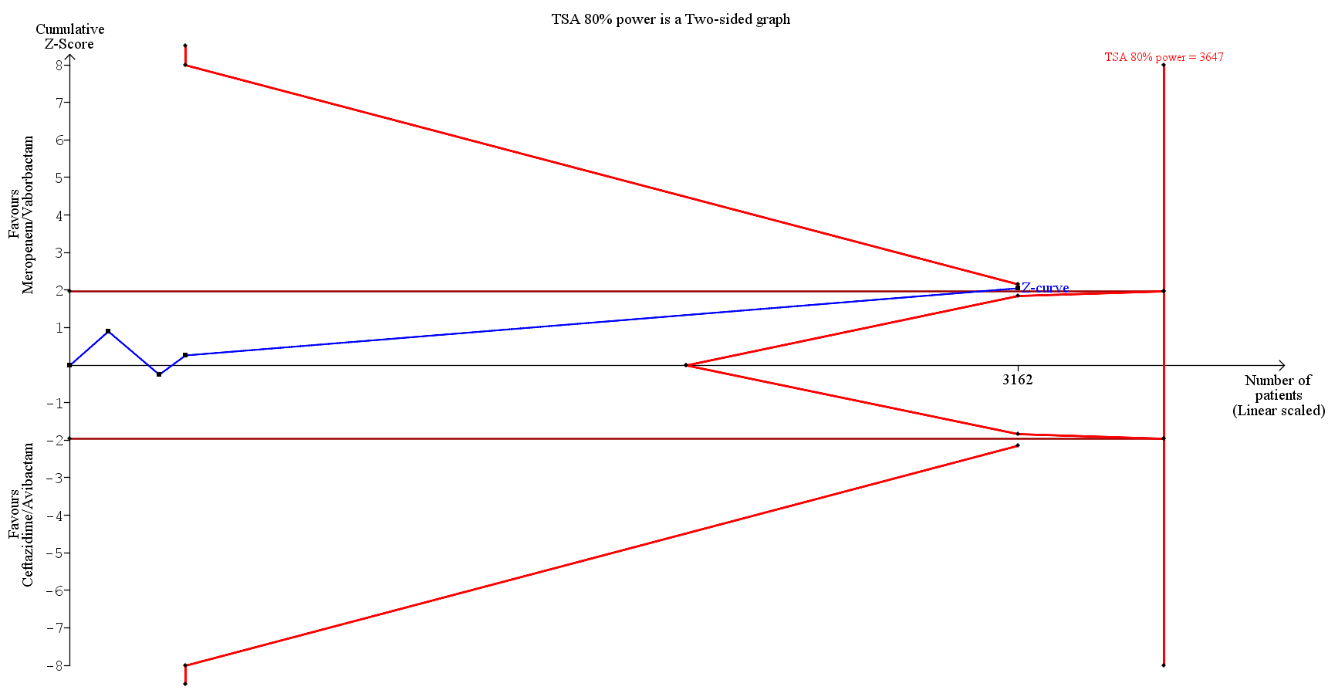


**Supplementary Figure 4**


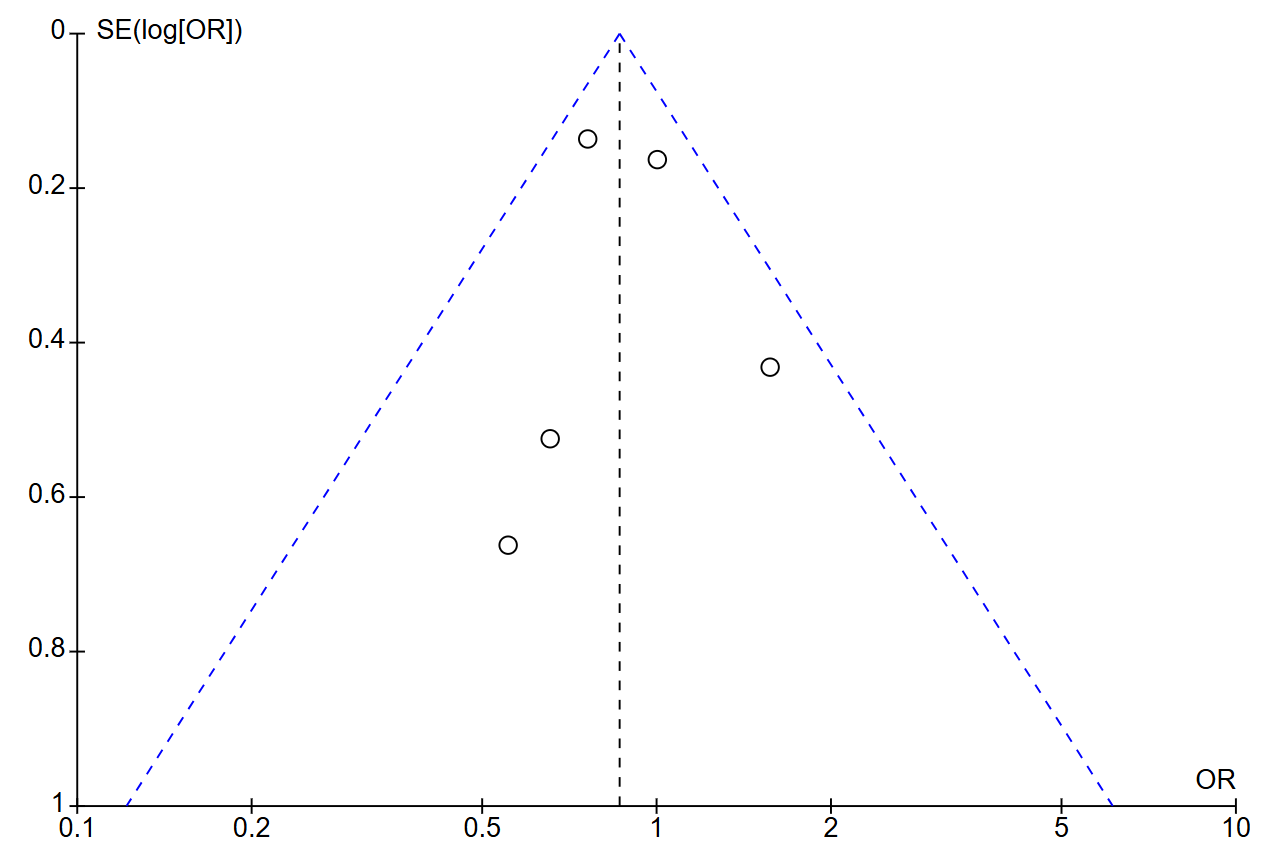


**Supplementary Figure 5**
